# Accelerating Pneumococcal Conjugate Vaccine introductions in Indonesia: key learnings from 2017 to 2022

**DOI:** 10.1186/s40249-023-01161-5

**Published:** 2023-11-28

**Authors:** Anithasree Athiyaman, Putri Herliana, Atiek Anartati, Niken Widyastuti, Prima Yosephine, Gertrudis Tandy, Sherli Karolina

**Affiliations:** 1 Global Vaccines Delivery, Clinton Health Access Initiative, Boston, USA; 2 Country Office, Clinton Health Access Initiative, Jakarta, Indonesia; 3 Directorate of Immunization, Ministry of Health, Republic of Indonesia, Jakarta, Indonesia

**Keywords:** New vaccine introduction, Immunization, Indonesia, Pneumococcal conjugate vaccines, Sustainability, Coverage, Gavi

## Abstract

Despite high pneumococcal disease and economic burden in Indonesia and interest to introduce pneumococcal conjugate vaccine (PCV), there were challenges in establishing a comprehensive strategy to accelerate and enable the introduction in country in the early 2010s. Starting in 2017, Clinton Health Access Initiative and partners supported the government of Indonesia with evidence-based decision-making and implementation support for introducing PCV into the routine immunization program. Indonesia has since accelerated PCV roll out, with nationwide reach achieved in 2022. On the path to PCV introduction, several challenges were observed that impacted decision making on whether and on how to optimally roll out PCV, resulting in significant introduction delays; including (1) a complex country context with a devolved government structure, fragmented domestic funding streams, and an imminent transition out of major immunization donor (Gavi) support; (2) strong preference to use domestically sourced products, with limited experience accessing global pooled procurement mechanism including for vaccines; and (3) concerns around programmatic feasibility and sustainability. This case study documents key insights into the challenges experienced and how those were systematically addressed to accelerate new vaccine introduction in Indonesia, with support from local and global stakeholders over time. The learnings would be beneficial for other countries yet to introduce critical new vaccines, in particular those with similar archetype as Indonesia e.g., middle-income countries with domestic manufacturing capacity and/or countries recently transitioning out of Gavi support.

## Background

Though pneumonia remained the leading cause of post neonatal child mortality in Indonesia and introducing pneumococcal conjugate vaccine (PCV) could avert 7365 deaths per year [1], as late as 2016, the government of Indonesia experienced challenges in establishing a comprehensive strategy to accelerate the introduction of the PCV vaccine. Though intentions for introduction were included in the 2016–2020 comprehensive multiyear plans for immunization (cMYP) (and previous plans as early as 2007), there was no official confirmation from the Ministry of Health (MOH) on when and how this introduction will happen.

Challenges to introduce PCV were not unique. Pathways to introduce other new life-saving vaccines such as rotavirus (RV) vaccines, Japanese encephalitis (JE) vaccines and human papilloma virus (HPV) vaccines were also not clearly illustrated in the cMYP. Several barriers are attributed to these new vaccine introduction challenges, leading to significant delay in introduction and slow scale up once rolled out. These include a complex country context with a devolved government structure, fragmented domestic funding streams, and an imminent transition out of major immunization donor (Gavi, the Vaccine Alliance) support at the end of 2017 impacting financing capabilities and lack of adequate data and access to the evidence needed to inform the decision of whether and on how to optimally introduce PCV. There is also preference for local sourcing of vaccines and other health commodities in Indonesia, which is legally mandated by law with few exceptions, and limited experience in accessing global procurement mechanisms (e.g., via UNICEF Supply Division). Given there was no domestically manufactured product available for PCV, there were concerns around procuring and financing such vaccines.

Clinton Health Access Initiative (CHAI) started by engaging MOH Expanded Immunization Program (EPI) and PCV champions within MOH to facilitate regular coordination meetings of MOH, partners, and other key stakeholders like the Ministry of Planning towards accelerating decision-making on PCV introduction. Once a demonstration project was decided to start in two focal districts (2017), CHAI supported central EPI to develop a robust operational plan and roadmap for phased scale up. At the subnational level, CHAI provided targeted technical assistance such as by conducting readiness assessments to identify risks to a successful introduction, supporting trainings for health workers and village volunteers, providing recommendation post-launch for immediate course correction. This led to a high uptake of PCV, showcasing programmatic feasibility and increasing MOH confidence to scale up the program further. The coordination meetings became more intense to determine the cost-effectiveness and affordability of a scaled-up PCV program. This includes deliberation of different procurement modalities that can give the country the most value for its money. The coordination meetings also helped instill ownership and secure stakeholders buy in on programmatic and financial support (budget allocation) to implement the program.

## Key learnings in accelerating PCV decision making and implementation

The challenges experienced can be categorized into three main buckets:

1. Challenges in high-level decision making and corresponding financing for vaccine procurement and delivery
2. Limitations of existing procurement and distribution systems
3. Limited confidence in the programmatic feasibility and sustainability of PCV introduction

**Fig. 1:**
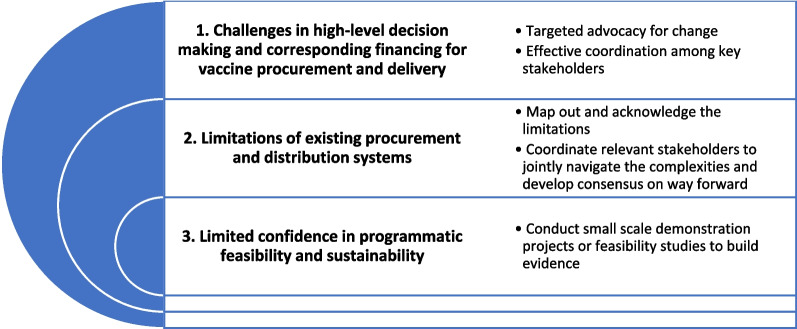
Key challenges experienced, and approaches undertaken to accelerate PCV decision making in Indonesia. *PCV* Pneumococcal conjugate vaccines

Each of these required a multi-stakeholder led differentiated approach to resolve the challenges. The section below outlines the learnings from the key approaches that were proven effective between 2017 and 2022 (see Fig. 1).

### Challenge 1: Challenges in high-level decision making and corresponding financing for vaccine procurement and delivery

#### Action: Practice targeted advocacy for change and effective coordination among key stakeholders

Indonesia was slated to transition out of Gavi support at the end of 2017 and had been ramping up co-financing obligations towards fully self- financing. The cost of vaccine procurement for antigens offered in the routine immunization program was already taking up the highest allocation (22%) of all MOH strategic preventive purchases [2]. While pneumonia was the leading cause of child death, procuring PCV for delivery nationwide will significantly increase the government’s vaccine procurement budget by three-fold [3]. There was significant hesitation among key decision makers on government capacities to allocate and disburse necessary funding to make this introduction possible. Given domestic funding allocations are done on an annual basis (note that this has since been revised, and multi-year planning and budget allocation for health is possible in country, and in nascent stages of implementation at the time of this publishing), there was also high a risk of funding not materializing on a timely basis year-on-year to sustain post-introduction. Indonesia also has a decentralized governance for health suggesting that operational costs for PCV implementation will need to be secured at the subnational level. Coordination between national and subnational stakeholders was fragmented, further adding to the challenges.

To mitigate this, CHAI and partners helped to map expanded set of stakeholders required for introduction decision making and supported with effective coordination. Upon identification of key decision makers, CHAI facilitated regular meetings with the same representatives each for accountability and follow up. CHAI also used the Gavi Transition timeline as a burning platform for the government to consider introducing PCV at a more affordable price while it is still accessible as a Gavi-eligible country.

CHAI then developed multi-year cost projections and annual plans to reflect the national PCV introduction strategy, and helped the MOH visualize gains by conducting a cost-effectiveness study [4]and developing advocacy decks tailored to different audiences. This showcased that PCV is highly cost-effective and also affordable if procured at a certain price. The advocacy materials also highlighted how PCV introduction can reduce overall child mortality, offset the costs of pneumococcal disease treatment endured by the National Health Insurance, and helped Indonesia achieve the Sustainable Development Goals (SDG). By fostering high level government ownership and engagement (within MOH including the Procurement Unit and Planning Bureau; but also across sectors including with the Ministry of Planning, Ministry of Finance, and Office of the President) MOH were able to secure the necessary buy-in and corresponding financial commitment. Targeted advocacy efforts were also conducted at the subnational level to ensure plans and budget for PCV introduction/ delivery activities could be secured ahead of introduction.

### Challenge 2: Limitations of existing procurement and distribution systems

#### Action: Map out and acknowledge the limitations, coordinate relevant stakeholders to jointly navigate the complexities and develop consensus on the way forward

Historically, vaccines procurement for the routine immunization program in Indonesia was conducted locally using the country’s e-catalogue system [5]. Indonesia has domestic vaccine manufacturing capacities i.e., through Biofarma, a state-owned pharmaceutical company that manufacturers and/or imports all vaccines in the EPI program. This strong local preference is mandated through a Ministerial decree, with small exceptions when Biofarma is not able to fulfill MOH request via local production and importation is needed. While this is primarily to ensure supply security as well as to promote scientific and economic development, the government had to decide whether to continue waiting for a domestic product to be ready or to start importing for public health reasons.

As a consequence, there was also limited experience up until then for the MOH in accessing UNICEF Supply Division (UNICEF SD) pooled procurement mechanisms for health commodity purchasing and poor use of global market intelligence on available products, pricing, supply availability and reliability [6]. PCV products were imported and locally available in the private fee-based health service facilities, however the price of which remained around 5 times higher than the global eligible price commitments (USD 20/dose compared to USD 3.5/dose). Hence, securing PCV supplies locally would be very inefficient and severely limit government capacities to introduce and scale up nationally.

Recognizing this challenge, stakeholder consultations were conducted to increase knowledge/awareness, assess the opportunities of procuring through different mechanism, and identify potential risks as well as implications. Through close coordination with global and country partners, CHAI supported the government to navigate complex legal and regulatory policies to execute the new procurement process and successfully access the Gavi Advanced Market Commitment (AMC) mechanism, ensuring advance payment and securing PCV vaccine supplies at affordable prices. Activities implemented include conducting cost-effectiveness analysis of PCV in Indonesia context, comparing UNICEF AMC price with government’s self-procurement price (USD 162 vs USD 747 per QALY gained^1^); developing cost-projection analysis for accurate estimate of budgetary needs and affordability; facilitating capacity building for MOH staffs on procurement and market intelligence; engaging intensely stakeholders beyond MOH, e.g., Office of the President to increase awareness on the high-impact of PCV program to reduce child mortality and position Indonesia well in reaching SDGs, Ministry of Finance to showcase cost-saving potential of pneumonia prevention through PCV program vs. pneumonia treatment incurred by the National Health Insurance; as well as initiating regular coordination with local manufacturer to understand timeline for potential domestic product so MOH can make informed decision for introduction. In collaboration with UNICEF, CHAI also supported in planning logistics and handling to distribute vaccines to subnational cold chain points.

### Challenge 3: Limited confidence in the programmatic feasibility and sustainability of PCV introduction

#### Action: Conduct small scale demonstration projects or feasibility studies to build evidence

Despite strong global evidence, some important stakeholders still needed to be convinced of the benefits of the introduction and its operational feasibility within the context of Indonesia. And concerns were also expressed around country capacities to achieve high coverage for new vaccines, given that the last (and only) new vaccine introduced in recent years was the first dose of Inactivated Polio Vaccine (IPV) in 2016 (note that second dose of Measles and Rubella MR2 vaccine was rolled out between 2017–2018, but unlike IPV this was a switch from MCV2 and in that sense less complex) which reached 66% coverage, thirteen percentage points lower than Penta3 coverage, despite being offered at the same time-point.

**Fig. 2:**
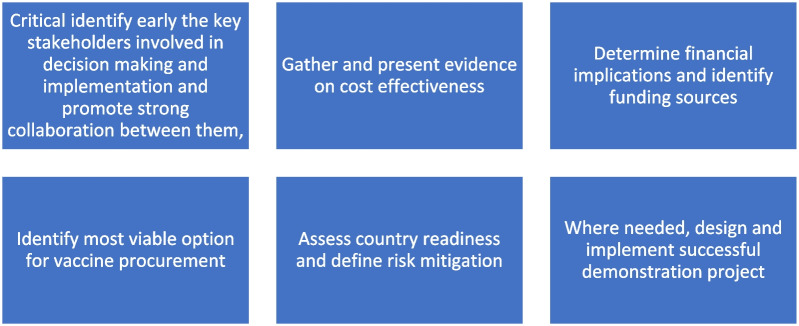
Key recommendations from Indonesia’s PCV decision making experience. *PCV* Pneumococcal conjugate vaccines

To alleviate these, CHAI supported the MOH in executing a demonstration project for PCV in select areas to assess programmatic feasibility. During which, best practices based on experience in other countries were implemented to ensure strong introduction process, e.g., carrying out readiness and post-launch assessments, identifying cold chain capacity gaps. In West and East Lombok where PCV was introduced in 2017, the coverages of PCV 1 and 2 are comparable to well-established Penta 1 and 2, and all coverages are above 80% (see Fig. 2).

## Conclusions

The lessons learned from Indonesia around evidence-informed decision making, strong coordination and project management resulted in accelerated national PCV introduction in country, with all provinces and districts introducing PCV vaccine in the public system by September 2022. The following are identified as the key enablers, and shared below as recommendations for other global partners and country stakeholders to adopt for their use cases: critical to identify early the key stakeholders involved in decision making and implementation and promote strong collaboration between them, gather and present evidence on cost effectiveness; determine financial implications and identify funding sources, identify the most viable option for vaccine procurement, assess country readiness and define risk mitigation and where needed, design and implement successful demonstration project.

## Data Availability

Generated based on work conducted in Indonesia, primary data and secondary sources from government reports and other program related documentation.

## Abbreviations

CHAI: Clinton health access initiative
CMYP: Comprehensive multi year plan
EPI: Expanded immunization program
IPV: Inactivated Polio Vaccines
JE: Japanese Encephalitis Vaccines
HPV: Human Papilloma virus Vaccines
MOH: Ministry of Health
PCV: Pneumococcal conjugate vaccines
RV: Rotavirus vaccines
UNICEF: UN Children’s Fund
